# New challenges of fetal therapy in Japan

**DOI:** 10.1111/jog.15320

**Published:** 2022-06-08

**Authors:** Seiji Wada, Katsusuke Ozawa, Haruhiko Sago

**Affiliations:** ^1^ Center for Maternal‐Fetal, Neonatal and Reproductive Medicine National Center for Child Health and Development Tokyo Japan

**Keywords:** congenital diaphragmatic hernia, critical aortic stenosis, fetal therapy, low urinary tract obstruction, myelomeningocele

## Abstract

**Aim:**

To review new challenges of fetal therapy in Japan after the establishment of four existing fetal therapies as standard prenatal care with National Health Insurance coverage over the past 20 years.

**Methods:**

Reported studies and our current research activities related to four fetal therapies newly performed in Japan were reviewed.

**Results:**

Fetoscopic endoluminal tracheal occlusion (FETO) for congenital diaphragmatic hernia (CDH) aims to occlude the trachea using a detachable balloon to promote lung growth. Following the recent successful completion of an international randomized controlled trial for CDH, in which we participated, FETO is offered for severe left CDH to perform balloon insertion at 27–29 weeks and removal at 34 weeks of gestation. Fetal cystoscopy (FC) for low urinary tract obstruction was introduced to overcome the demerits of vesicoamniotic shunting. FC may provide a proper diagnosis by visual observation of the urethra and physiological treatment of the posterior urethral valve. The effectiveness of open fetal surgery for myelomeningocele (MMC), direct surgery with laparotomy and hysterotomy, for ameliorating hindbrain herniation and the motor function was demonstrated, but it was also associated with substantial maternal and fetal risks. Fetal aortic valvuloplasty (FAV), ultrasound‐guided fetal aortic balloon dilation for critical aortic stenosis with evolving hypoplastic left heart syndrome may improve left heart development and maintain biventricular circulation. Feasibility and safety studies for FC, MMC open fetal surgery, and FAV are currently ongoing.

**Conclusions:**

Clinical research on FETO, FC, MMC open fetal surgery, and FAV has proceeded with careful preparations in Japan.

## Introduction

Fetal therapy is medical intervention for the fetus in utero. It is performed to rescue fetuses at risk of perinatal death or severe irreversible damage, despite optimal management after birth. Fetal therapy was first attempted in the 1960s when the first fetal intrauterine blood transfusion (IUT) was performed.^1^ Technologies to detect precise fetal conditions were not well advanced at that time. Advances in ultrasound imaging in the late 1970s, which enabled prenatal diagnostics, led to active attempts in fetal therapy. The concepts of “fetus as a patient” and “unborn patient” were advocated. With the accumulation of experimental and clinical experience in fetal treatment, the first conference of the International Fetal Medicine and Surgery Society (IFMSS) was held in 1982.^2^ It noted that “Treatment of the fetus with a potentially correctable defect is promising but still experimental. All case material should be reported, regardless of outcome, to a fetal‐treatment registry.” This was recognized as the beginning of the modern fetal therapy.

Under ultrasound guidance, IUT for fetal anemia,^3^ vesicoamniotic shunting (VAS) for fetal low urinary tract obstruction (LUTO),^4^ and thoracoamniotic shunting (TAS) for fetal hydrothorax^5^ were performed in the 1980s. Later, fetal aortic valvuloplasty (FAV) for critical aortic valve stenosis (CAS)^6^ and radiofrequency ablation (RFA) for twin reversed arterial perfusion (TRAP) sequence^7^ were also performed. Open fetal surgery, direct surgery on the fetus with hysterotomy, was performed for LUTO,^8^ congenital diaphragmatic hernia (CDH),^9^ and congenital pulmonary airway malformation^10^ by pediatric surgeons in the US Fetoscopic laser surgery (FLS) for twin‐twin transfusion syndrome (TTTS) was started in 1990^11^ and it thereafter developed into a minimally invasive percutaneous procedure that was recognized as the most successful fetal therapy.^12^, ^13^, ^14^ A fetoscopic approach, which later developed as fetoscopic endoluminal tracheal occlusion (FETO), replaced open fetal surgery for CDH.^15^, ^16^ There are two trends in fetal therapy, one is moving from invasive open surgery to less invasive fetoscopic techniques, while the other is moving from clinical descriptions and retrospective analysis to randomized controlled trials.^17^ Following a successful clinical trial,^18^ open fetal surgery for myelomeningocele (MMC) continues.

In Japan, several attempts at fetal therapy, including IUT,^19^ VAS,^20^ and FLS by laparotomy for TTTS,^21^ were reported in the 1980–1990s. Actual Japanese clinical research of fetal therapy began in the early 2000s with the start of FLS program for TTTS. We reported good outcomes of our initial experience of FLS for TTTS at four centers.^22^ Our results confirmed that FLS was the optimal treatment option for TTTS at 16–25 weeks of gestation. They also led to the coverage of costs by Japan National Health Insurance from April 2012. FLS was the first fetal therapy to be reimbursed by Japan National Health Insurance.^23^ With our favorable findings, FLS has also been applied for triplets,^24^ TTTS at 26–27 weeks^25^, ^26^ and selective intrauterine growth restriction with oligohydramnios.^27^, ^28^ With the introduction of the Solomon technique and the accumulation of experience, we have achieved a very high survival rate (the at least one survival rate was >98%)^29^ with favorable long‐term neurodevelopmental outcomes for TTTS treated by FLS.^30^ TAS was the second fetal therapy to be compensated by Japan National Health Insurance, with the favorable results of our prospective one‐arm trial using a “double‐basket catheter” (Hakko Co., Nagano, Japan).^31^ We also reported the effects of TAS on primary hydrothorax and on trisomy 21 based on a nationwide survey.^32^, ^33^ Owing to two studies,^34^, ^35^ RFA for TRAP sequence has become the third fetal therapy to be approved for coverage by Japan National Health Insurance. We have clarified the good long‐term outcomes of TRAP sequence after RFA.^36^ FLS, TAS, and RFA are minimally invasive fetal therapies that have been recognized as standard prenatal care in Japan.^37^ In addition, in 2020, IUT, which remains the oldest type of fetal therapy, was the fourth fetal therapy to be approved for coverage by Japan National Health Insurance based on the findings of recent studies.^38^, ^39^

In the past 20 years, we have been engaged in clinical research of existing fetal therapies in Japan to establish them as standard prenatal care with the approval of National Health Insurance coverage. As a result, FLS, TAS, RFA, and IUT have become standard prenatal treatments in Japan. We have proceeded to the next step to challenge new fetal therapies, including FETO for CDH, fetal cystoscopy (FC) for LUTO, open fetal surgery for MMC, and FAV for CAS. The current status of fetal surgical therapy in Japan is shown in Table 1. This review will focus on our ongoing experience of feasibility and safety studies and clinical trials for these new fetal therapies.

**TABLE 1:** Current fetal surgical therapy in Japan

**1. Fetal therapy covered by National Health Insurance (approved year)**
Fetoscopic laser surgery for twin‐to‐twin transfusion syndrome (2012)
Thoracoamniotic shunting for fetal hydrothorax (2012)
Radiofrequency ablation for twin reversed arterial perfusion sequence (2019)
Intrauterine blood transfusion for fetal anemia (2020)
**2. Fetal therapy for which clinical trials have been completed**
Fetoscopic endoluminal tracheal occlusion for congenital diaphragmatic hernia
**3. Fetal therapy for which feasibility and safety studies have started**
Fetal cystoscopy for lower urinary tract obstruction
Fetal open surgery for meningomyelocele
Fetal aortic valvuloplasty for critical aortic stenosis

## FETO for CDH

**FIGURE 1:**
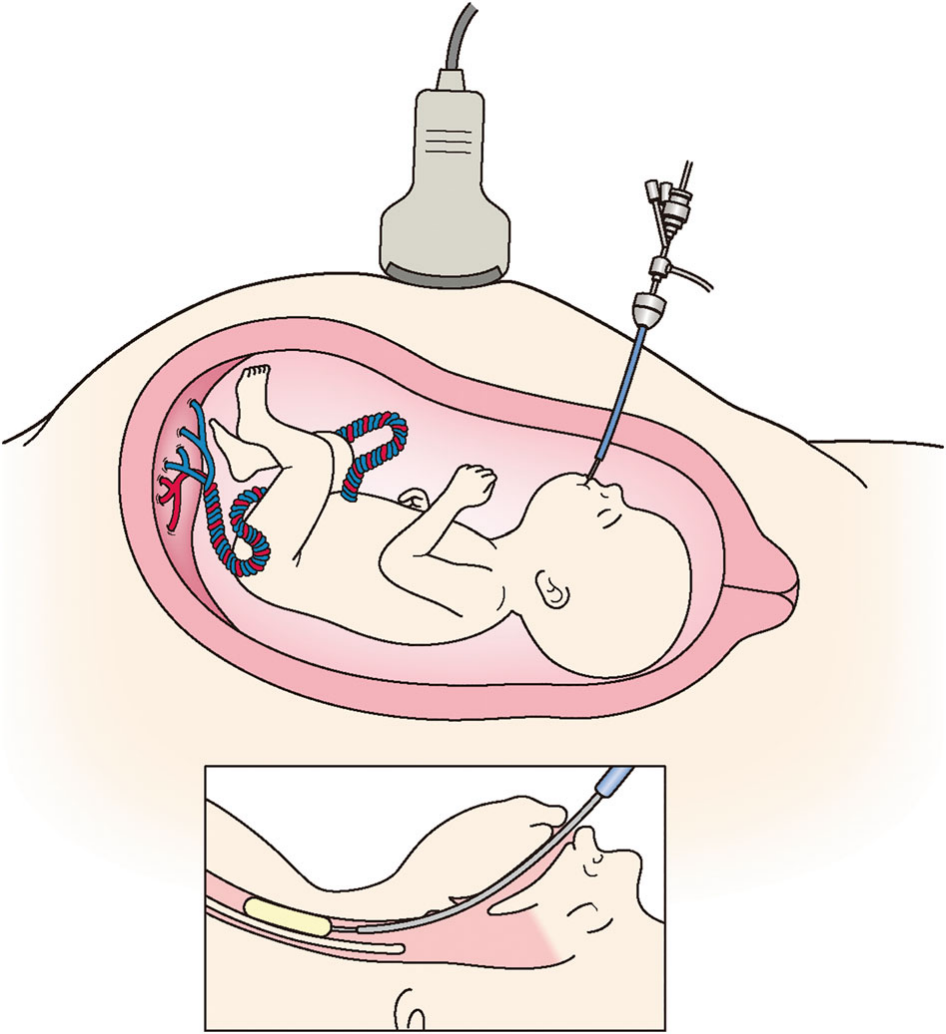
A schematic representation of fetoscopic endoluminal tracheal occlusion for congenital diaphragmatic hernia. A balloon is placed in the trachea through a fetoscope

CDH is a congenital disease characterized by herniation of the abdominal organs into the thoracic cavity through a diaphragm defect.^40^, ^41^, ^42^ The prevalence of CDH is approximately 1 in 4000 live births. The overall survival rate has increased with advances in prenatal diagnostics that permit referral to experienced high volume centers, and advances in postnatal managements, especially in respiratory care. The survival rate of prenatally diagnosed isolated CDH was 79% in a nationwide survey in Japan.^43^ However, the outcomes of patients with severe CDH remain poor, which denotes the limitation of postnatal treatment. Direct diaphragm repair of the fetus with laparotomy was unsuccessfully attempted,^9^ then the treatment strategy moved from anatomic repair to physiological manipulations.^44^ The major pathology of CDH is impairment of normal pulmonary development caused by intrathoracic herniation of the abdominal viscera. It was observed that fetal tracheal ligation promoted lung growth^45^ and experimentally reversed pulmonary hypoplasia in CDH.^46^ Fetal tracheal occlusion is thought to cause the accumulation alveolar fluid, which resulted in lung enlargement. Following an attempt at clipping of the trachea,^47^ a surgical technique to implant a detachable balloon into the trachea under a fetoscope, named FETO, was established (Figure 1).^15^, ^16^

Harrison et al. reported a randomized controlled trial of FETO using a 5‐mm uterine port with laparotomy for left CDH with liver herniation and a lung‐to‐head ratio (LHR) of <1.4 in 2003.^15^ The results showed no improvement in survival with FETO. With many preterm birth cases in the FETO group and high survival rates in the control groups, there could be some room to improve surgical techniques and patient selection. Later, although it was a retrospective study, the European FETO Study Group reported that FETO using a percutaneous approach with a 3.3 mm cannula in left CDH with LHR <1.0 was associated with a significant survival advantage in a study population that was limited to more severe cases in comparison to previous studies.^48^, ^49^ These findings led to an international randomized controlled trial for CDH called the Tracheal Occlusion To Accelerate Lung Growth (TOTAL) trial.

In Japan, we initially performed clinical research on the natural history of prenatally diagnosed CDH for the selection of candidates for fetal therapy.^50^, ^51^, ^52^ We showed that the combination of liver and stomach positions was useful to differentiate prognoses in left CDH.^52^ Later, we conducted a feasibility and safety study for severe left CDH cases of Kitano G3 stomach position with intrathoracic liver herniation from 2013 to 2016.^53^ Balloon insertion was performed at 27–31 weeks, and balloon removal was performed at 34 weeks. Balloon insertion was successful in all 11 cases, and balloon removal was performed at 34 weeks in all 10 cases, with the exception of 1 case that resulted in fetal death. The rate of survival to discharge was 45% (5/11), and there were no serious adverse maternal events, although there was one fetal death associated with cord strangulation. This study showed that FETO was feasible without maternal morbidity in Japan.

The TOTAL trial consisted of a protocol for severe left CDH (defined as an observed‐to‐expected LHR [o/e LHR] of <25%) to improve survival and a protocol for moderate left CDH (defined as an o/e LHR of ≥25.0% to ≤34.9%, irrespective of liver position, or an o/e LHR of ≥35.0% to ≤44.9% with intrathoracic liver herniation) to improve pulmonary complications. Balloon insertion was performed at 27–29 weeks for severe CDH and at 30–31 weeks for moderate CDH. Balloon removal was performed at 34 weeks for both severe and moderate CDH. From 2018, we participated in the TOTAL for severe CDH, which was started in 2011 and stopped early at 2020 as its efficacy was demonstrated after the third interim analysis, which included 80 women. The results for severe cases showed a significantly improved survival to discharge rate, of 40% in the FETO group in comparison to 15% in the expectant care group.^54^ The rates of survival to 6 months of age were identical to the rates of survival to discharge. In contrast, the TOTAL trial for moderate CDH, which involved 196 women, started in 2008 and was completed in 2019, failed to show a significant benefit of FETO.^55^ The rate of survival to discharge in moderate CDH was 63% in the FETO group and 50% in the expectant group (relative risk [RR]: 1.27. 95% confidence interval [CI]: 0.99–1.63). The rate of survival to 6 months without oxygen supplementation was 54% in the FETO group and 44% in the expectant group (RR: 1.2. 95% CI: 0.93–1.65). However, the analysis of pooled data, including these two randomized controlled trails suggests that FETO increases survival for both moderate and severe CDH.^56^ Late balloon insertion for moderate CDH in the TOTAL trial is a point to be considered, although early balloon insertion increases the risk of preterm delivery. In Japan, FETO is currently offered for severe isolated left CDH (o/e LHR <25%) to perform balloon insertion at 27.0–29.9 weeks (Table 2). The balloon is removed at 34 weeks of gestation using a fetoscopic procedure, ultrasound guided puncture or ex utero intrapartum treatment. Our center is the only center in Japan that performs FETO. The establishment of one or two more additional FETO centers, reconsidering the indication criteria (including some moderate cases), and to obtaining National Health Insurance coverage are tasks to be accomplished for FETO in Japan.

**TABLE 2:** Our criteria for performing FETO

Single pregnancy 27.0–29.9 weeks of gestation Left congenital diaphragmatic hernia o/e LHR <25% No other severe congenital malformations

Abbreviations: FETO, fetoscopic endoluminal tracheal occlusion; o/e LHR, observed‐to‐expected lung‐to‐head ratio.

## FC for LUTO

The prevalence of LUTO is approximately 1 in 5000 births.^57^ LUTO is a congenital obstructive abnormality of the urethra that could cause renal failure and bladder dysfunction. Severe cases with oligohydramnios in the first or early second trimester are associated with pulmonary hypoplasia and high neonatal mortality.^58^, ^59^ The characteristic ultrasound findings of LUTO are enlarged bladder (megacystis) and hydronephrosis. The etiology of LUTO includes posterior urethral valves (PUV), urethral stenosis or atresia, persistent cloaca, Prune‐belly syndrome, and megacystis microcolon intestinal hypoperistalsis syndrome. The differential diagnosis of these conditions in utero is usually difficult.

Fetuses with severe LUTO with oligohydramnios face a very poor prognosis, then fetal treatment is considered. Vesicoamniotic shunting (VAS), which continuously drains urine from the bladder to the amniotic cavity has been performed to prevent renal dysfunction and pulmonary hypoplasia in utero. In Japan, VAS has been performed using a double‐basket catheter.^20^ The PLUTO study, the only randomized controlled trial of VAS, which was stopped due to poor recruitment, showed that the VAS group had a higher 2‐year survival rate in comparison to the expectant group, but there were no significant differences in the postnatal renal function.^60^ A systematic review for LUTO showed that the rate of survival to 6 months of age was significantly higher in the VAS group in comparison to the expectant group, but there were no significant differences in 2‐year survival or in the postnatal renal function.^61^ These results indicate that there is little evidence to support that VAS improves fetal long‐term outcomes, including the renal function.

Recently, we reported a retrospective study of 87 cases of prenatally diagnosed LUTO that were managed at two Japanese centers over 15‐year period.^62^ More than half of the cases were terminated before 22 weeks of gestation and only eight cases (9%) underwent VAS. The liveborn rates in the VAS group and the conservative management group were 100% (8/8) and 56% (18/32), respectively (*p* = 0.034). In these groups, 6‐month survival with a normal renal function was achieved in 38% (3/8) and 16% (5/32), respectively (*p* = 0.608). VAS is suggested to be effective for achieving perinatal survival. PUV, a common cause of LUTO, is treatable after birth but can lead to the development of renal and pulmonary dysfunction in utero. Then, PUV is thought to be a good candidate for fetal therapy. PUV was found in one‐fifth of the cases with a confirmed diagnosis. However, most PUV cases were managed expectantly which resulted in a poor prognosis. An accurate prenatal diagnosis of LUTO, which enables the detection of PUV in utero is crucial for improving the outcomes of LUTO. Considering the limitations of ultrasound diagnosis and VAS, a new diagnostic and management strategy for LUTO is expected.

To overcome the limitations associated with ultrasound and VAS in the diagnosis and treatment of LUTO, FC was introduced in 1990s.^63^ The expected advantages of FC are as follows: (1) the proper diagnosis of the etiology of LUTO by visual observation in the fetal bladder; and (2) direct treatment of the etiology which can provide more physiological urinary drainage than VAS. An accurate prenatal diagnosis could lead to appropriate counseling for patients, which will allow them to consider pregnancy outcomes. FC could treat PUV by opening the membrane using laser ablation. Ruano et al. reported a multicenter case control study of 111 fetuses with severe LUTO. The survival rates at 6 months of age in the FC, VAS, and no intervention groups were 13/34 (38%), 7/16 (44%), and 12/61 (20%), respectively. In the FC, VAS, and no intervention groups, a normal renal function was achieved in 12/16 (75%), 6/10 (60%), and 11/28 (39.3%) cases, respectively. Both FC and VAS will improve 6‐month survival; however, FC is the only treatment that may prevent renal impairment.^64^ Sananes et al. reported a retrospective study of 50 LUTO cases in which FC was performed. The etiology of LUTO was diagnosed in 32/35 (91%) cases. At 2 years of age, the survival rate of patients with PUV was 54% (15/28), and 73% (11/15) showed a normal renal function.^65^ FC can provide an accurate diagnosis of the cause of LUTO and the specific fetal treatment with an improved prognosis. In contrast, there was a negative report that did not show the superiority of FC in terms of long‐term outcomes, probably due to inadequate visualization of the posterior urethra.^66^ FC is expected to be a promising fetal therapy; however, studies on FC have been limited.

**FIGURE 2:**
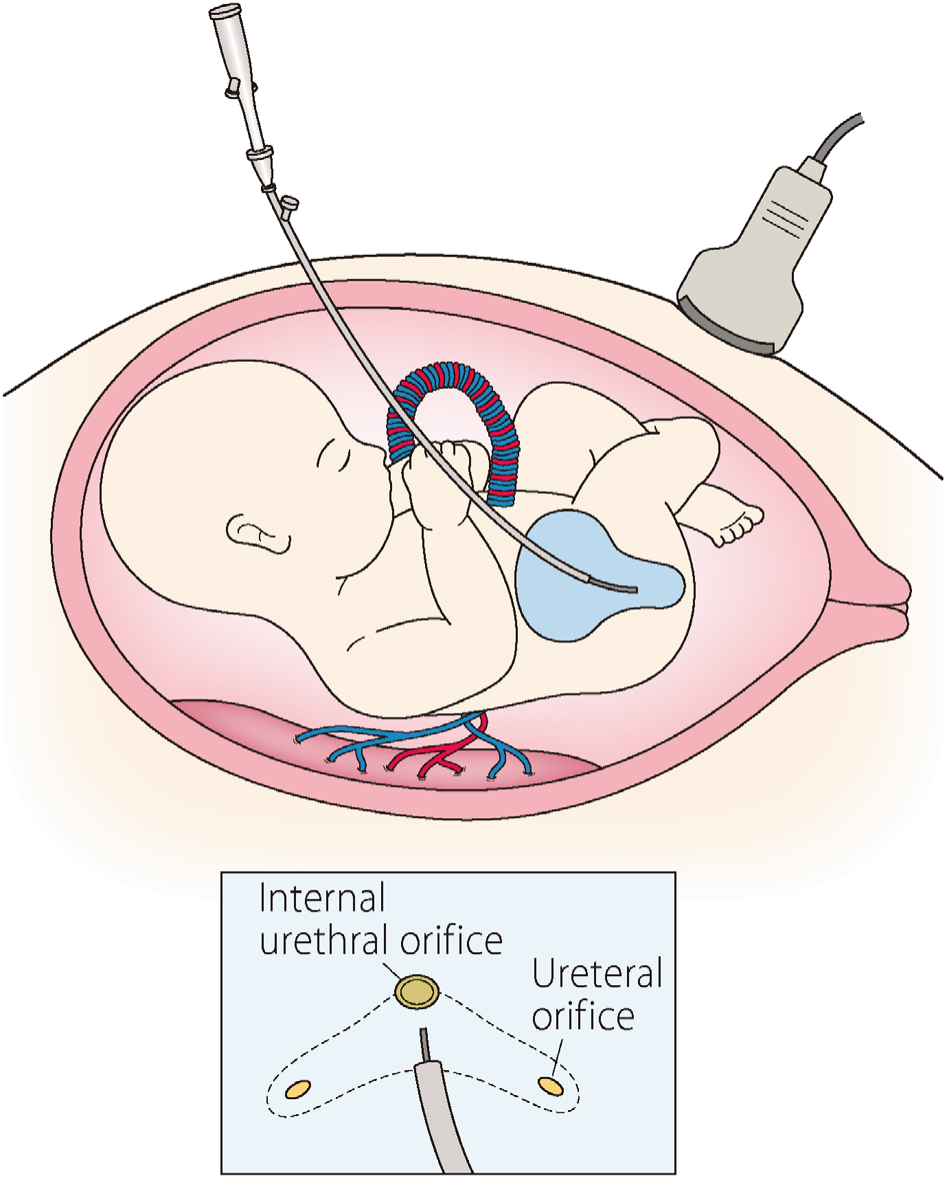
A schematic representation of fetal cystoscopy for fetal low urinary tract obstruction. To visualize the urethra and ureteral orifice and to open the valve using a laser in a case of posterior urethral valve

It is necessary to examine the benefits of FC for fetuses with LUTO. We are currently conducting a safety and feasibility study of FC for the fetal diagnosis and treatment of LUTO at the Osaka Maternal and Child Medical Center and our center. The indication criteria for FC are shown in Table 3.^67^ FC is performed by inserting a 1.0–1.3 mm fetal cystoscope (Karl Stortz, Tuttlingen, Germany) into the fetal bladder through the maternal abdomen under ultrasound guidance (Figure 2). Etiologies including urethral atresia or stenosis, PUV, and others are examined by observation of the internal urethral orifice from inside the bladder with investigation of the passage of water through the urethra. In the case of PUV, laser ablation using an Nd‐YAG laser is performed. This study is a single‐arm clinical trial registered as UMIN000043128 (http://www.umin.ac.jp).

**TABLE 3:** Our criteria for performing FC

Singleton pregnancy 16.0–25.9 weeks of gestation Severe LUTO defined as the presence of a megacystis on ultrasound Fetal urine β‐2 microglobulin value below the reference level (6.3–11.2 mg/L)* Re‐accumulation of urine in the bladder after vesicocentesis No renal cystic changes No other severe congenital malformations

Abbreviations: FC, fetal cystoscopy; LUTO, fetal low urinary tract obstruction. and *, reference 67.

## Fetal Surgery for MMC

MMC is a common type of spina bifida in which the spinal cord protrudes into a sac that is not covered by the skin through the spinal defect. The incidence of MMC is estimated to be 0.2–0.3 per 1000 live births in Japan.^68^ At the lesion of MMC, the spinal cord is damaged, resulting in peripheral nerve injury distal to the lesion. The lesions are often located in the lumbar or sacral vertebrae resulting in limb disabilities, and bladder and rectal dysfunction. In addition, Chiari malformation type II, which is characterized by hindbrain herniation is present in almost all cases of MMC. The brainstem and cerebellum tend to herniate into the vertebral cavity due to decreased spinal cord space pressure, resulting in impairment of the circulation of cerebrospinal fluid and the central respiratory function. Children who develop hydrocephalus may have neurodevelopmental impairment. Postnatal surgery consists of repair of the meningocele early in life and ventriculo‐peritoneal shunting (V‐P shunt) for hydrocephalus afterwards. With appropriate postnatal treatment, including surgery, children with MMC are still burdened with irreversible peripheral and central neurological damage.

The pathogenesis of MMC has been proposed to consist of two stages of neurological damage, referred to the “two‐hit theory.” The “first hit” is the primary developmental abnormality due to failed closure of the neural tube. The “second hit” is the secondary neural abnormality due to chemical damage from chronic exposure to amniotic fluid and mechanical damage from contact with the uterus. The principle of fetal surgery is to reduce irreversible neural damage by preventing neural injuries caused by the “second hit.”^69^ Furthermore, fetal surgery is expected to improve the hindbrain herniation, Chiari malformation type II, by suppressing the reduction of the spinal cord cavity pressure during the fetal period. Fetal surgery for MMC is the first example of the application of fetal intervention for a nonlethal condition.

Research on fetal treatment of MMC has mainly been conducted in the United States since the 1990s. In 1995, Meuli‐Simmen et al. reported that a lumbar spinal cord meningocele was surgically formed in a fetal sheep and repaired in utero to preserve the limb function.^70^ In 1998, Tulipan and Bruner performed MMC repair with laparotomy and hysterotomy in four patients at 28–32 weeks of gestation and showed recovery of hindbrain herniation after birth.^71^ In 1998, Adzick et al. performed fetal surgery with laparotomy and hysterotomy for thoracic to sacral MMC at 23 weeks of gestation, which resulted in the rescue of Chiari malformation type II and good developmental milestones.^72^ More than 200 cases of fetal surgery for MMC were performed between 1997 and 2003. Observational studies suggested that fetal surgery could reverse hindbrain herniation with a reduced need for postnatal V‐P shunt, and improve the lower limb and bladder function.^73^, ^74^, ^75^ They also showed increased maternal complications, including preterm PROM, preterm labor, and uterine dehiscence at the uterotomy site.

To investigate the safety and efficacy of prenatal repair of MMC, a randomized controlled trial of prenatal versus postnatal surgery for fetal MMC, named the Management of Myelomeningocele Study (MOMS), was conducted at three centers in the United States between 2003 and 2010.^18^ The inclusion criteria for the study were MMC with the upper boundary located at Th1‐S1, hindbrain herniation, and gestational age 19–25 weeks. Prenatal surgery significantly reduced the composite incidence of fetal or neonatal death and V‐P shunting in comparison to postnatal surgery (68% vs. 98%, RR: 0.70, 95% CI: 0.58–0.84). In particular, the actual rate of V‐P shunting in the prenatal‐surgery group was significantly lower in comparison to the postnatal‐surgery group (40% vs. 82%, *p* < 0.001). Prenatal surgery was also associated with improved mental development and motor function at 30 months, hindbrain herniation by 12 months and ambulation by 30 months. However, prenatal surgery was accompanied by an increased incidence of preterm delivery, uterine incision defects, and maternal blood transfusion. MOMS shows that fetal surgery for MMC is effective for reducing V‐P shunting and improving the motor function, but is associated with substantial maternal and fetal risks.

**FIGURE 3:**
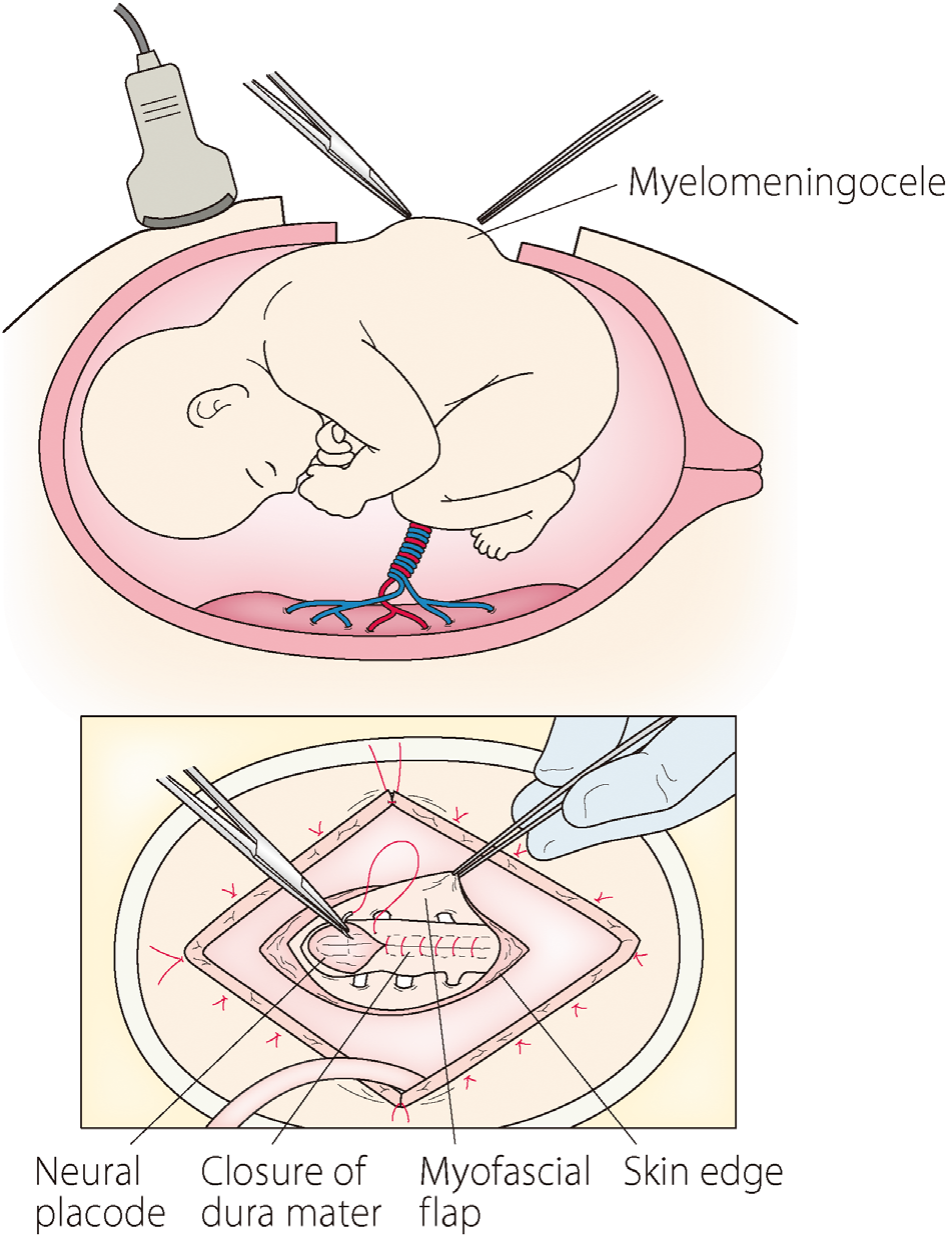
A schematic representation of open fetal surgery for myelomeningocele. The prolapsed neural placode is closed with dura mater and further covered with a myofascial flap and skin

Although open fetal surgery using laparotomy and hysterotomy has rarely been performed outside the United States, the successful results of MOMS stimulated the introduction of fetal surgery for MMC. We experienced only one case of open fetal surgery for congenital pulmonary airway malformation.^76^ We started a feasibility and safety study of open fetal surgery for MMC at Osaka University Hospital and our center in 2020. The study is sponsored by the Japan Agency for Medical Research and Development and is registered as UMIN000040088 (http://www.umin.ac.jp). The indications and procedures are as same as those for MOMS (Table 4, Figure 3). We are facing difficulties in enrollment. Our nationwide survey showed that more than half of fetal MMC cases were diagnosed after 26 weeks of gestation, which is over the limit of fetal surgery and three‐quarters of cases diagnosed before 22 weeks of gestation chose termination of pregnancy.^77^ In addition to knowledge of fetal surgery as a treatment option, the earlier detection of MMC by obstetric ultrasound is essential.

**TABLE 4:** Our criteria for performing open fetal surgery for MMC

Singleton pregnancy 19.0–25.9 weeks of gestation MMC with upper boundary located at T1‐S1 Hindbrain herniation No severe congenital malformations not related to MMC Spinal kyphosis <30°

Abbreviation: MMC, myelomeningocele;

## FAV for CAS

Many congenital heart diseases can be prenatally diagnosed by fetal ultrasound. Most of structural heart diseases do not actually vary during gestation. However, some congenital heart defects could change dramatically as gestation advances, and may benefit from fetal intervention. The evolution of fetal (CAS) with enlarged left ventricle in mid‐gestation to hypoplastic left heart syndrome (HLHS) at term was reported.^78^ It is thought that increased afterload due to left ventricular outflow tract stenosis leads to hypertrophy, later atrophy of the left ventricular myocardium. HLHS is characterized by mal‐development of the left‐sided structures, which hampers biventricular circulation. Advances in cardiac surgical management contribute to the improved prognosis of children with HLHS, although they still have a high risk of mortality and morbidity.^79^, ^80^, ^81^ Fetal cardiac intervention which could improve fetal hemodynamics and may prevent the progression of mal‐development of the left heart structures.

If left ventricular outflow tract stenosis can be relieved in utero, there is a possibility of improving the development of the left ventricle and maintaining biventricular circulation after birth. Fetal cardiac intervention can be considered for CAS with evolving HLHS. Fetal cardiac intervention for CAS was first reported in 1991 by Maxwell et al. who performed ultrasound‐guided fetal aortic balloon dilation of the aortic valve in two fetuses with CAS.^6^ These first two cases resulted in fetal or neonatal death. In 1995, Allan et al. reported the first case of survival.^82^ In the initial 12 cases reported in the 1990s, more than half of the cases faced technical failure and only one case survived.^83^ The establishment of a fetal intervention program at two centers, namely Boston Children's Hospital (USA) and The Women's and Children's Hospital Linz (Austria), in 2000 marked a turning point in fetal cardiac intervention.^84^

The Women's and Children's Hospital Linz reported a total of 103 cases of CAS with evolving HLHS who underwent FAV between December 2001 and September 2020. Eighty‐seven percent of the cases were technically successful and 89% of fetuses were liveborn with a biventricular rate of 55%.^85^ Boston Children's Hospital reported 143 fetuses with CAS with evolving HLHS who underwent FAV between 2000 and 2017. Eighty‐four percent of cases were technically successful; the fetal death rate was 8%. Fifty percent of the liveborn children eventually achieved biventricular circulation.^86^ Taken together, approximately a technical success of 80%, fetal death rate of 10%, and biventricular circulation rate of 50% are expected following FAV for CAS with evolving HLHS.

**FIGURE 4:**
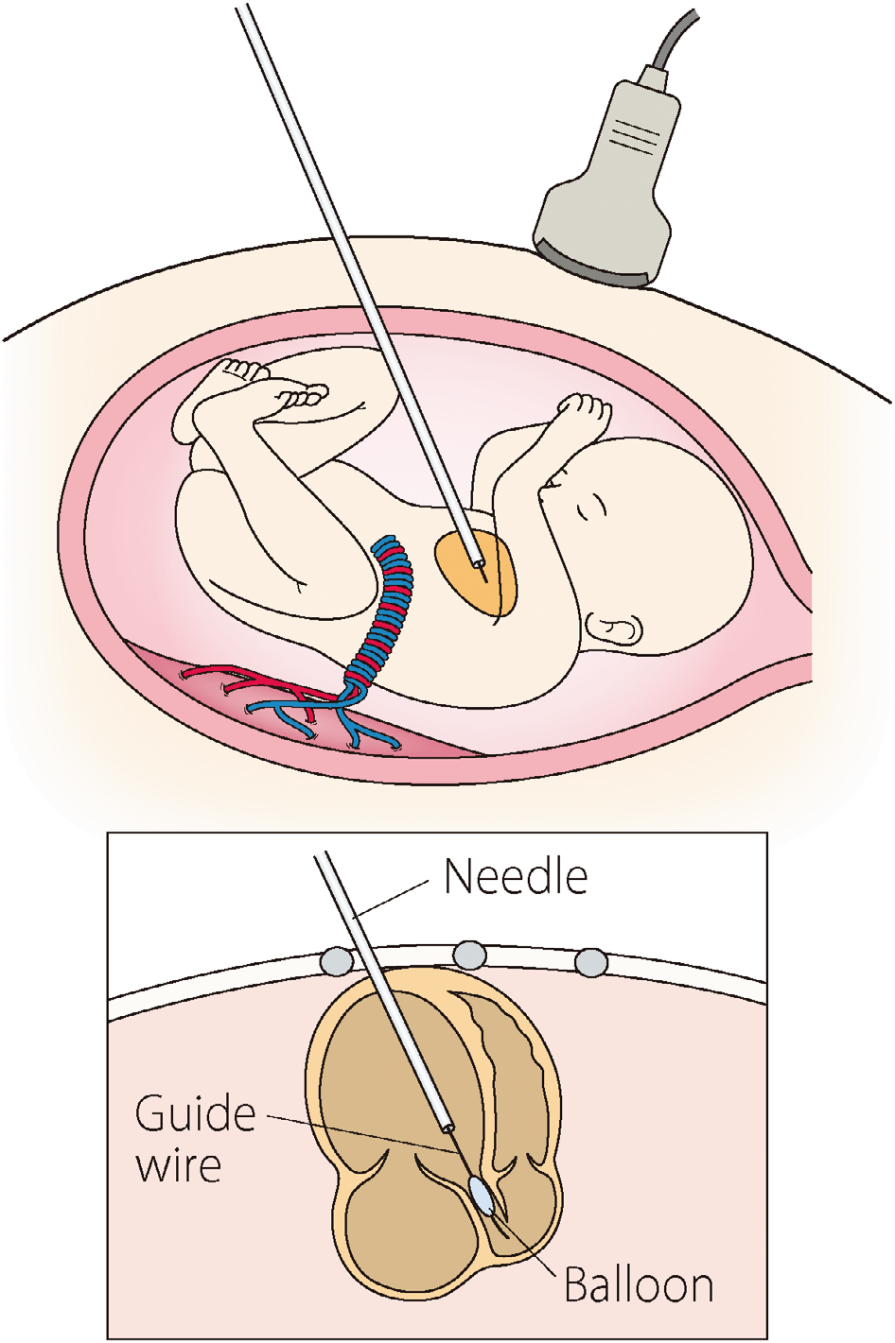
A schematic representation of fetal aortic valvuloplasty for critical aortic valve stenosis. Ultrasound‐guided dilation of the aortic valve with a guidewire and balloon

To start the program of FAV for CAS with evolving HLHS in Japan, we conduct a feasibility and safety study on collaboration with the Japanese Society of Fetal Cardiology and Japanese Society of Pediatric Cardiology and Cardiac Surgery. The patient selection criteria consist of key echocardiographic findings to estimate the potential for evolving HLHS and a biventricular outcome (Table 5).^87^ With consensus of a panel of pediatric cardiologists, FAV is performed at our Center. Under fetal anesthesia, an 18‐gauge needle is inserted into the fetal left ventricle through the thorax under ultrasound guidance. A coronary balloon catheter is introduced into the needle and placed in the aortic annulus with wire guidance and is inflated to dilate the aortic valve (Figure 4). This study is registered as UMIN000036649 (http://www.umin.ac.jp).

**TABLE 5:** Our criteria for performing FAV

Singleton pregnancy 22.0–31.9 weeks of gestation Valvular aortic stenosis including all of the following: Decreased mobility of aortic valve leaflets Antegrade Doppler color blood flow jet across aortic valve smaller than valve annulus diameter No or minimum subvalvular LV outflow obstruction LV function qualitatively depressed Retrograde or bidirectional flow in the transverse aortic arch or two of the following: Monophasic mitral inflow Doppler pattern Left‐to‐right flow across atrial septum or intact atrial septum Bidirectional flow in pulmonary veins LV long‐axis Z core > −2 Threshold score ≥4 (≥4 of the following) LV long‐axis Z score >0 (1 point) LV short‐axis Z score >0 (1 point) Aortic annulus Z score >−3.5 (1 point) Mitral valve annulus Z score > −2 (1 point) Mitral regurgitation or aortic outflow peak systolic gradient ≥20 mmHg (1 point) No other severe congenital malformations

Abbreviations: FAV, fetal aortic valvuloplasty; LV, Left ventriculus.

## Conclusion

In Japan, new challenges for fetal therapy include FETO for CDH, FC for LUTO, open fetal surgery for MMC, and FAV for CAS, following the establishment of existing fetal therapies, and including FLS, TAS, RFA, and IUT, as standard prenatal care with National Health Insurance coverage. With the recent successful completion of an international randomized controlled trial for CDH, in which we participated, FETO is offered as a clinical treatment for severe isolated left CDH. FC for LUTO, open fetal surgery for MMC and FAV for CAS are currently being evaluated in ongoing feasibility and safety studies. We hope that these new therapies will contribute to the progress of fetal therapy, which could benefit unborn patients.

## Author Contributions

All authors revised the manuscript, approved the manuscript to be published, and agree to be accountable for all aspects of the work in ensuring that questions related to the accuracy or integrity of any part of the work are appropriately investigated and resolved.

## Conflict of interest

None declared.

## Data Availability

Data sharing is not applicable to this article as no new data were created or analyzed in this study.
